# A Bioinformatics Toolkit for Next-Generation Sequencing in Clinical Oncology

**DOI:** 10.3390/cimb45120608

**Published:** 2023-12-04

**Authors:** Simon Cabello-Aguilar, Julie A. Vendrell, Jérôme Solassol

**Affiliations:** 1 Montpellier BioInformatics for Clinical Diagnosis (MOBIDIC), Molecular Medicine and Genomics Platform (PMMG), CHU Montpellier, 34295 Montpellier, France; 2 Laboratoire de Biologie des Tumeurs Solides, Département de Pathologie et Oncobiologie, CHU Montpellier, Université de Montpellier, 34295 Montpellier, France; j-vendrell@chu-montpellier.fr (J.A.V.); j-solassol@chu-montpellier.fr (J.S.)

**Keywords:** bioinformatics, clinical oncology, targeted therapy, pipeline, NGS, SNV, CNV, MSI

## Abstract

Next-generation sequencing (NGS) has taken on major importance in clinical oncology practice. With the advent of targeted therapies capable of effectively targeting specific genomic alterations in cancer patients, the development of bioinformatics processes has become crucial. Thus, bioinformatics pipelines play an essential role not only in the detection and in identification of molecular alterations obtained from NGS data but also in the analysis and interpretation of variants, making it possible to transform raw sequencing data into meaningful and clinically useful information. In this review, we aim to examine the multiple steps of a bioinformatics pipeline as used in current clinical practice, and we also provide an updated list of the necessary bioinformatics tools. This resource is intended to assist researchers and clinicians in their genetic data analyses, improving the precision and efficiency of these processes in clinical research and patient care.

## 1. Introduction

Progress in next-generation sequencing (NGS), including an increase in its accessibility and cost effectiveness, has enabled comprehensive genetic testing in many cancer centers and transformed cancer treatment. In particular, NGS has permitted the advancement of precision oncology focused on identifying genetic changes in tumors that include single-nucleotide variants (SNVs), copy number variations (CNVs), small insertions and deletions (indels), structural variants (SVs), and microsatellite instability (MSI) [1,2]. Such valuable insights into the molecular characteristics of tumors provided by NGS have made it an essential tool for the diagnosis and treatment of cancer [3].

Robust and reliable bioinformatics pipelines able to organize, interpret, and accurately identify these molecular alterations from within sequencing datasets are crucial in the treatment decision-making process. The robustness ensures that the pipeline can handle variations in the data and produce consistent results, while the reproducibility ensures that the same results can be obtained when the pipeline is run multiple times. In addition, the comprehensive traceability and understanding of how the pipeline works ensure that others are able to reproduce the results. To this end, a well-designed and well-documented bioinformatics pipeline can provide reliable and accurate guidance for oncologists.

In this review, we focus on the role of bioinformatics in NGS-based precision oncology. Specifically, we explore the bioinformatics steps involved in this process, including the calling of genetic alterations, their annotation, and interpretation. To provide a practical example of how each step is implemented, we describe a typical bioinformatics pipeline and reporting workflow for targeted sequencing analysis of solid tumors.

Of note, we have specifically focused this review on the analysis of data from Illumina sequencing, given its widespread adoption in the scientific community. It is noteworthy that various sequencing platforms with unique strengths and applications are available. For instance, Oxford Nanopore Technologies offers long-read sequencing, providing valuable insights into structural variations. Pacific Biosciences (PacBio) is recognized for its ability to generate long reads, facilitating the resolution of complex genomic regions. A thorough understanding of the strengths and limitations of different platforms is essential for making informed choices when implementing a NGS bioinformatic pipeline in clinical oncology. While Illumina is extensively utilized, readers are encouraged to assess their specific needs and explore alternative platforms that may better align with their objectives.

## 2. Workflow Management

**Figure 1:**
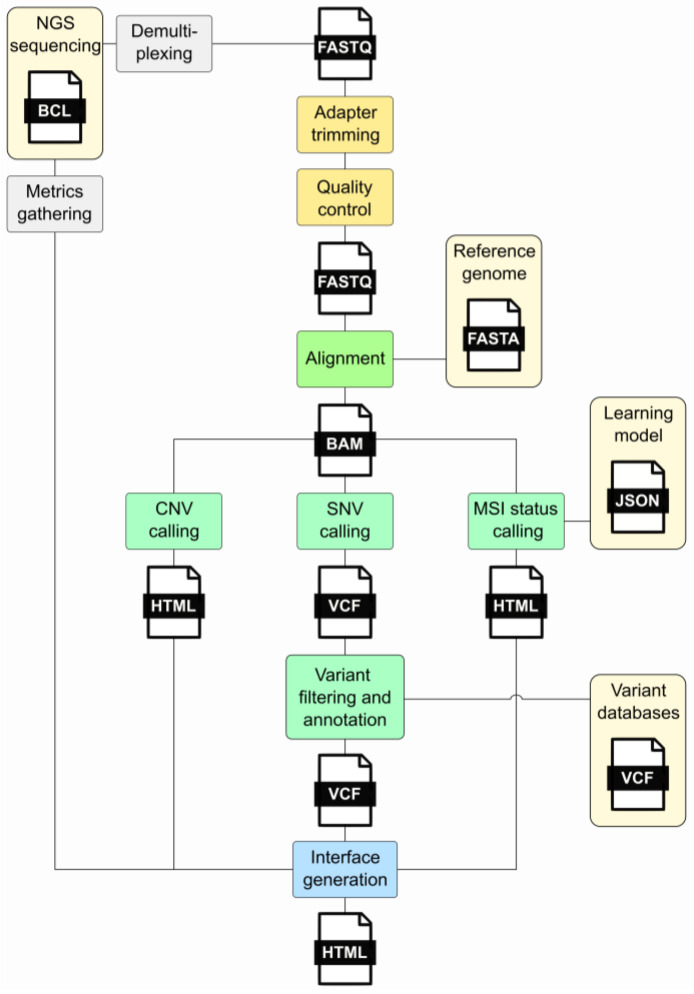
Major steps of an NGS bioinformatics pipeline. This diagram illustrates the processes forming the pipeline and the files generated during its execution. The gray segments denote processes that exist independently of the pipeline. Light yellow signifies external prerequisites, while yellow represents the initial pipeline stages involving FastQ processing. The alignment stage is highlighted in green, while light green indicates the analyses conducted, encompassing SNV, CNV, and MSI status calling. The final step, interface generation, is illustrated in blue. Acronyms: FASTQ—a text-based file storing nucleotide sequences and corresponding quality scores; BAM—Binary Alignment Map; VCF—Variant Call Format; CNV—Copy Number Variation; SNV—Single-Nucleotide Variant; MSI—Micro Satellite Instability.

In clinical oncology, the rapid evolution of high-throughput sequencing technologies has increased data generation, necessitating robust and efficient bioinformatic pipelines for analysis. Command-line tools [4,5] offer a flexible and efficient means to handle these data. These tools enable bioinformaticians to construct intricate pipelines that encompass various stages of analysis. The command-line interface, with its text-based interaction, allows for precise control over parameters, facilitating the customization and optimization of workflows to suit the specific requirements of clinical oncology research. However, command-line tools rely solely on text-based interfaces, requiring users to input commands in a terminal or console, while workflow management tools commonly provide users with a graphical or text-based interface to design workflows, offering a more visually intuitive experience. Workflow management tools [6] also ensure the automation and standardization of the bioinformatics process and allow the user to define the order, parameters, and input data for a sequence of tools. They directly take care of the correct execution and documentation of the intermediate steps. Several workflow managers are available, including Snakemake and Nextflow, among others [7,8,9,10,11]. Such systems help bioinformaticians save time, reduce errors, and ensure the accuracy and reliability of their analyses. In cancer genomics, a bioinformatics pipeline is executed by the workflow manager such as that described in Figure 1 and comprises different steps: (i) quality control, (ii) adapter trimming, (iii) alignment, (iv) variant calling, (v) variant annotation, (vi) variant filtering, (vii) CNV calling, (viii) MSI status calling, and (ix) interface generation.

An up-to-date compilation of available tools for each step of the pipeline is provided in Table 1. It is important to mention that the Broad Institute provides a Genome Analysis Toolkit (GATK) [12], which contains a wide variety of tools designed for variant discovery and genotyping that covers the steps described in Figure 1. Moreover, the nf-core community project [13] has assembled a curated collection of analysis pipelines constructed with Nextflow including a somatic variant calling workflow, SAREK [14,15], available at “https://nf-co.re/sarek/3.4.0 (accessed on 1 December 2023)”. Nf-core offers portable and reproducible analysis pipelines and the support of an active community.

Galaxy [16] and Taverna [17] are both noteworthy platforms in the field of bioinformatics analysis. Galaxy, as an open-source platform, offers a web-based interface for analyzing high-throughput genomics data, especially NGS data. It accommodates users with varying levels of bioinformatics expertise, allowing them to create, execute, and share workflows for diverse bioinformatics analyses. Featuring a user-friendly graphical interface, Galaxy is accessible to a broad audience, providing tools and workflows for tasks such as sequence alignment, variant calling, and various genomic analyses. The platform emphasizes reproducibility, enabling users to systematically save and share their analyses. Taverna serves as a distinct workflow management system designed for various scientific applications, including bioinformatics. It facilitates the design and execution of workflows, providing a flexible environment for scientific analysis and automation. Additionally, Tavaxy [18] shortens the workflow development cycle by incorporating workflow patterns to streamline the creation process. It facilitates the reuse and integration of existing (sub-) workflows from Taverna and Galaxy, while also supporting the creation of hybrid workflows.

Noteworthy, private solutions also exist. For example, the DRAGEN secondary analysis pipeline ensures all the steps from sequencing files to annotated and filtered genetic alterations. It was recently benchmarked, and the authors claim its value in a preprint that came out this year [19].

**Table 1:** List of commonly used bioinformatic tools.

Process	Tools	References
Workflow managers	Nextflow, Snakemake	[7,8]
Quality control	fastp, FastQC *, Picard, MultiQC	[20,21,22,23]
Adapter trimming	fastp, trimmomatic, cutadapt *, BBDuk	[20,24,25,26]
Reads alignment	BWA *, Bowtie, HISAT2, STAR	[27,28,29,30]
Variant calling	HaplotypeCaller, freebayes, mutect2, verdict *	[31,32,33,34]
Variant filtering	dbSNP, 1000G, GnomAD *	[35,36,37]
Variant annotation	VEP *, MobiDetails, ANNOVAR, SnpEff	[38,39,40,41]
CNV calling	CNV-LOF, CoverageMaster, CNV-RF, DeepCNV, CNV_IFTV, HBOS-CNV, CNV-MEANN, ControlFREEC, ifCNV *, mcna	[42,43,44,45,46,47,48,49,50,51]
MSI status calling	MIAmS *, MSIsensor, MSIdetect, deltaMSI	[52,53,54,55]

* Used in our in-house bioinformatics pipeline.

## 3. FastQ Processing

### 3.1. Quality Control

**Figure 2:**
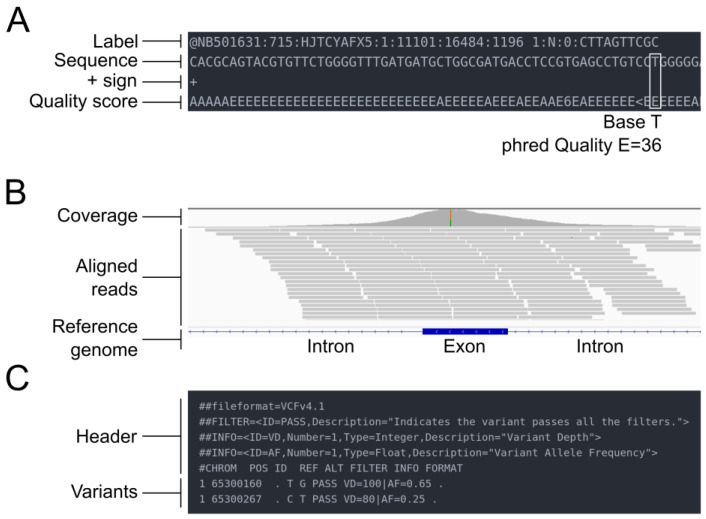
Overview of the different file types mentioned in the pipeline. (**A**) FASTQ file. (**B**) SAM/BAM file. (**C**) VCF file.

**Figure 3:**
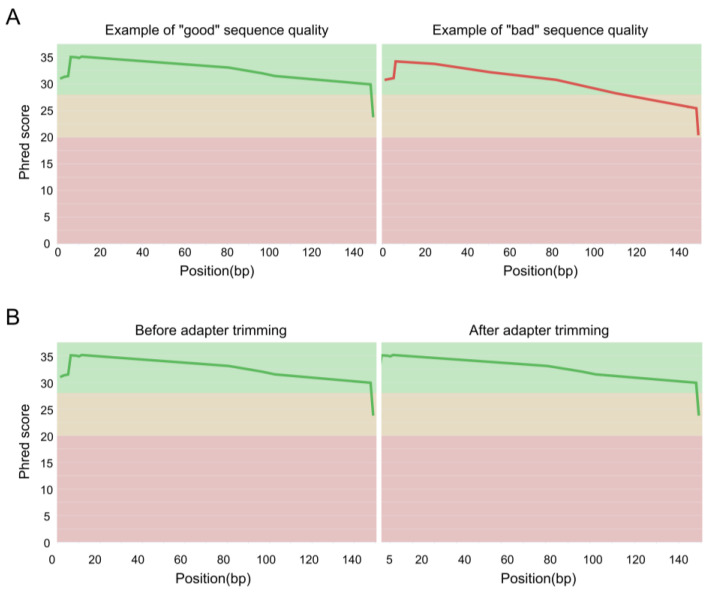
FastQC mean quality scores. (**A**) Examples of “good” and “bad” sequence quality. (**B**) Overview of the adapter trimming impact.

NGS sequencing produces binary base call sequence files (BCL) that are demultiplexed into FASTQ format sequencing files for each sample. The FASTQ format is a text-based format designed to store nucleotide sequences, along with their corresponding quality scores (Figure 2A). The initial stage of all bioinformatics pipelines is to assess the quality of the data. Indeed, sequence quality control is an essential step in the analysis of NGS data, which are generated in large volumes and can be prone to various types of errors, such as sequencing errors, adapter contamination, and sample cross-contamination. Sequence quality control aims to ensure that the sequencing data are accurate, reliable, and free from technical artifacts that could affect downstream analysis. It aims to identify low-quality bases, sequence bias, and over-representation of certain sequences. Quality assessment can be performed using tools such as fastp [20] or FastQC [21], a flexible and widely used tool for quality control, developed at the Babraham Institute to assess the quality of sequencing data in fastq files. This tool is robust, can be used on all operating systems, and offers both a graphical user interface and a command line interface. It is commonly incorporated by bioinformaticians as a quality control step in customized pipelines. The latest versions of FastQC include Picard [22], a tool developed by the Broad Institute that can manage SAM, BAM, and VCF files and perform quality control at different stages of the bioinformatics pipeline. An example of good and bad sequence quality profiles (i.e., the mean quality value across each base position in the read) obtained using FastQC is provided in Figure 3A. Moreover, MultiQC [23] consolidates data from various QC tools to create a cohesive report, complete with interactive plots, spanning multiple samples.

### 3.2. Adapter Trimming

Another preprocessing step is the adapter trimming, which involves removing adapter sequences, low-quality reads, and contaminating sequences from the raw sequencing data. The most widely used tools for data preprocessing are fastp [20], Trimmomatic [24], Cutadapt [25], and BBDuk [26]. In Figure 3B, we present quality profiles obtained using FastQC, illustrating the impact of adapter trimming with Cutadapt.

## 4. Alignment of the Nucleotide Sequence on a Reference Genome

After adapter trimming, the next step is to align the reads to a reference genome. The Genome Reference Consortium introduced the latest human reference genome, GRCh38 [56], in 2017, followed by subsequent improvements, the latest being GRCh38.p14 in March 2022, which remarkably reduced the number of gaps in the assembly to 349 compared to the initial version’s approximately 150,000 gaps. Notably, these gaps were predominantly found in regions like telomeres, centromeres, and long repetitive sequences. Last year, the Telomere-to-Telomere (T2T) Consortium presented the first fully assembled reference genome [57], T2T-CHM13, eliminating all gaps.

The alignment step is performed by read mapper software, which assigns a location on the reference genome to each read based on its sequence. Since the reads do not contain information about their location in the genome, the mapper infers this information by comparing the read sequence to the reference genome. Essentially, it checks which parts of the reference genome match the sequences in the reads, determining where these reads originated in the genome. However, this seemingly straightforward task is computationally intensive and time-consuming because the software must meticulously compare each read to the entire reference genome and assign a precise position for it. The computational demand arises from the need for high accuracy and reliability in determining the origin of each read, a fundamental step in understanding the genetic information contained within the sequenced sample. There are many different read mappers available, each with its own strengths and weaknesses. Common examples include BWA [27] for genome and Bowtie2 [28] for transcriptome. These tools employ a Burrows–Wheeler transform, a computational method invented by Michael Burrows and David Wheeler in 1994. This method involves rearranging character strings into sequences of similar characters, which offers significant computational benefits. Indeed, strings with repeated characters are easily compressible using techniques like move-to-front transform and run-length encoding. Various aligners employ distinct strategies; for instance, HISAT2 [29] is a graph-based genome alignment tool. The utilization of a graph-based approach allows leveraging theoretical advancements in computer science, resulting in a rapid and memory-efficient search algorithm. In transcriptome alignment, STAR [30] is also widely employed, using the Maximal Exact (Unique) Match concept for seed searching, it proves particularly advantageous for aligning long reads (>200 bp), such as those generated by third-generation sequencing.

The results of the read mapping step are usually provided in SAM format files, which can be converted to BAM format for more efficient storage and processing. SAM/BAM files can be accessed through the Integrative Genomics Viewer (IGV), allowing visualization of the reads (Figure 2B). The BAM files undergo different modifications during the alignment post-processing step, which includes tasks such as sorting, marking duplicate reads, and recalibrating base quality scores. The goal of these post-processing steps is to improve the accuracy and reliability of the final variant calls.

After the read mapping step, the resulting SAM/BAM files are sorted according to their genomic coordinates. This sorting is important because downstream analysis often relies on the order of the aligned reads. PCR duplicates are then commonly removed using tools such as Picard [22,58] or SAMtools [5]. PCR duplicates are identical copies of the same genomic fragment and can be introduced during sample preparation and PCR amplification steps. They can bias the analysis and lead to overrepresentation of certain regions of the genome. However, it is important to note that duplicated reads can also be biological copies originating from the same genomic location of chromosomes of different cells. For deep-coverage targeted sequencing approaches the probability of a duplicate read to be a biological copy increases with coverage, and therefore, the removal of duplicates is typically not performed in these cases.

## 5. Genetic Alterations Detection

### 5.1. SNV Calling

Variant calling is the critical step in identifying DNA alterations such as SNV or indels. This process involves comparing the DNA sequence of a sample (e.g., tumor tissue) to a reference genome or another sample from the same individual (e.g., normal tissue or blood). By detecting differences between the two sequences, variants can be identified. This is also a computationally intensive and time-consuming step, as the algorithms must compare each base to the reference. To perform this analysis, specialized software tools called variant callers are utilized. Called variants are usually stored in Variant Call Format (VCF) files. They consist of a header with various metadata, along with eight mandatory data columns, each row corresponding to a unique variant (Figure 2C).

Numerous variant callers are available, consolidating various statistical methods for variant detection. Noteworthy among them are GATK’s variant callers, HaplotypeCaller [31] and UnifiedGenotyper [59]. It is worth mentioning that with the transition from GATK3 to GATK4, UnifiedGenotyper was discontinued as HaplotypeCaller demonstrated superior performance, outperforming it across various metrics [60]. Also, among widely used variant callers for somatic variant calling are FreeBayes [32], mutect2 [33], and VarDict [34]. Those variant callers were benchmarked using synthetic datasets [61] and differences in true positives were minor, but the number of false positives could vary significantly. FreeBayes and VarDict exhibited notably higher false positives, despite VarDict also having the highest number of true positives. A joint approach, combining several variant callers outperforms individual tools, showing increased specificity, balanced accuracy, and fewer false positives [62,63]. However, it is worth noting that each variant caller generates a distinct VCF file with its unique nomenclature. To combine outcomes from multiple variant callers on the same sample, custom-made scripts are necessary. However, the appropriate choice of variant caller depends on the data type and the biological problems addressed. For further information regarding somatic variant calling algorithms, interested readers may consult the latest reviews [64,65].

### 5.2. Variant Filtering

In the context of somatic variant calling, germline variants and polymorphisms, must be filtered. To that end, the variants found in the tumor sample are compared to a database of known germline variants, such as dbSNP [35], 1000 Genomes Project [36] or GnomAD [37]. Any variants present in this database are likely to be germline variants and are filtered out. The remaining variants are considered potential somatic variants and undergo further analysis. This approach is not as reliable for rare variants or variants in poorly annotated regions of the genome. Furthermore, these algorithmic solutions for identifying somatic mutations have limitations, especially given the Eurocentric bias of many population-based allele frequency databases. Accuracy may be diminished for underrepresented minorities, where allele frequency data are more limited.

Another approach consists in using a normal control sample, involving the sequencing of DNA from both the tumor sample and a sample of normal tissue from the same patient, such as blood or normal tissue adjacent to the tumor. The variants identified in the normal sample are then compared to the variants identified in the tumor sample. Variants that are present in the tumor sample but not in the normal sample are considered potential somatic variants. This approach has higher specificity, but it requires sequencing of both tumor and normal samples, which increases the cost and complexity of the analysis.

### 5.3. Variant Annotation

Variant annotation is the process of compiling pertinent information to make informed decisions about a given variant, while minimizing the amount of manual parsing required. This includes basic annotations such as the affected gene, whether it is in a coding or noncoding region, and whether it is synonymous or nonsynonymous. This step can be conducted by various software including VEP [38], AnnoVar [40], or SnpEff [41], for example. Additionally, more complex annotations such as clinical significance can also be included. The clinical significance of a variant holds particular importance for clinicians as it can aid in the decision making regarding patient care, including treatment options and risk assessment. The classification of variants is generally based on their association with specific diseases or phenotypes and includes categories such as pathogenic, likely pathogenic, of unknown significance, likely benign, or benign. However, the classification of variants may differ among various databases and tools, which can result in difficulties when interpreting and comparing results obtained from different sources of information. For instance, ClinVar [66], a freely accessible and public archive of reports links particular variants to known functional or clinical features, or the *TP53* Database that compiles *TP53* variant data reported in the published literature since 1989 [67]. Similarly, the database offered by the ENIGMA consortium provides annotations for *BRCA1/2* and *CHEK2* [68]. In contrast, other tools, such as SIFT [69] or Polyphen [70], categorize variants based on their in silico predicted impact on protein function. Recently, Chen et al. introduced AlphaMissense [71], an adaptation of AlphaFold [72], a neural network-based model, specifically designed for predicting missense variant pathogenicity. AlphaMissense demonstrated superior performance with an area under the receiver operator curve (auROC) of 0.940, evaluated on 18,924 ClinVar test variants. It outperformed models that were not trained directly on ClinVar and even surpassed models trained directly on ClinVar data. The emergence of these tools highlights the evolving landscape of the field. Consequently, it is crucial to meticulously evaluate the sources of annotation data employed in variant interpretation. Recently, an aggregator called MobiDetails [39] was developed to provide comprehensive and up-to-date variant annotation. It displays the most pertinent annotation databases and in silico effect predictors in a single web page.

Online databases such as DGIdb [73], OncoKB [74], and CIViC [75] are commonly utilized for querying drug–gene interactions. These databases also function as robust resources for extracting insights into the potential diagnostic implications and prognostic value of identified variants. Such information can be particularly beneficial for physicians, enabling them to adapt therapeutics and optimize patient care. In addition to direct interactions, it would also be advantageous to annotate genes with indirectly interacting drugs, i.e., drugs that target proteins upstream or downstream of the gene within the relevant pathway. Of note, customized in-house databases can be utilized for variant annotation. For instance, annotating a variant if it has been previously observed in another patient or sequencing experiment can provide valuable insights.

### 5.4. CNV Calling

In clinical oncology, CNV as biomarkers can help predict how a patient will respond to specific therapies. For instance, several targeted therapies are FDA-approved for the treatment of breast cancer patients with ERBB2 amplification [76,77], while MET amplification in non-small-cell lung carcinomas is a known resistance mechanism to tyrosine kinase inhibitors [78,79]. As a result, incorporating CNVs into a laboratory pipeline is critical for improving patient outcomes. There exist three primary methods for identifying CNV from NGS data: read-pair (RP), split-read (SR), and read-depth (RD).

RP methods such as BreakDancer [80], compare the average insert size of sequenced read-pairs to an expected size based on a reference genome. Variations from the predetermined average insert size are used to detect gain or loss of genomic materials. Shorter or longer insert sizes compared to the expected size correspond to the loss or gain of materials, respectively. SR methods evaluate CNV using paired reads where only one read of the pair has a reliable mapping quality while the other one partially fails to map to the reference sequence. These discrepancies within a read pair can potentially provide the precise position of insertion/deletion events. Tools implementing SR strategies (e.g., SVseq2, Gustaf, PRISM [81,82,83]) enable the detection of these breakpoints but are limited to short insertions or deletions. The RD approach consists in counting the aligned reads overlapping a genomic region and comparing the read counts between the sample of interest and a reference to determine CNV. A local decrease or increase in sequencing depth will correlate to loss or gain/amplification of loci, respectively.

Numerous tools for CNV detection are available, employing diverse algorithms including artificial intelligence, intricate statistical modeling, and more [42,43,44,45,46,47,48,49]. Recent RD-based methods such as ifCNV [50] and mCNA [51] have demonstrated remarkable performance in clinical practice, offering both fast computational times and high sensitivity and specificity.

### 5.5. MSI Status Calling

MSI is a biomarker of DNA mismatch repair deficiency commonly observed in cancer [84]. Accurate determination of MSI status is important for prognostic and therapeutic purposes. For instance, MSI status can predict the response to immunotherapy in colorectal cancer [85]. Traditional methods for analyzing microsatellite status involve length distribution analysis of multiplex-PCR generated DNA fragments from tumor samples, which can be labor-intensive and time-consuming [86]. NGS technology offers an alternative method for MSI determination. NGS-based applications such as MIAmS [52], MSISensor [53], deltaMSI [54] or more recently the solution published by Sophia genetics, MSIdetect [55], can determine MSI status. It requires specific spiking of microsatellite loci in the targeted panel. This approach offers several advantages over traditional methods, including high accuracy and higher efficiency. MIAmS is a scalable application that does not require paired normal tissue for comparison and generates a user-friendly report for interpretation. The use of NGS-based applications for MSI determination is increasingly being adopted in clinical practice due to their improved performance and convenience.

### 5.6. Implementation of a Pipeline

Typically, developing a robust NGS analysis pipeline in clinical oncology demands a rigorous scientific approach. It is imperative for medical oncologists to clearly convey their requirements to both biologists and bioinformaticians who can propose effective solutions. It is important to note that any pipeline needs to be adjusted based on specific experimental conditions. Moreover, adapting the pipeline to the computing architecture is crucial for optimal performance. Additionally, specific variant filtering and annotation criteria can be established by the bioinformatician in collaboration with the medical oncologists, tailored to the biological problem being addressed.

For illustration purposes, we provided a list of tools used in our bioinformatics pipeline, and we expect it may aid those faced with numerous options (Table 1). The selection of tools was guided by subjective considerations including the ease of implementation, the utilization in other pipelines for computing harmonization and inter-pipeline compatibility, and a proven track record in efficiently handling large volumes of clinical samples. All the tools mentioned in this review are regularly maintained and kept up to date. It is essential for individuals considering the implementation of a pipeline in their laboratory to consult the documentation of each tool, as each tool has its unique strengths and weaknesses. In recent years, best practices for the implementation of a bioinformatic pipeline have been published [87]. Physicians and bioinformaticians seeking to implement a new pipeline should familiarize themselves with this literature.

## 6. Future Developments

Moving forward, further developments in bioinformatics are crucial for the advancement of clinical oncology. These ongoing efforts aim to address emerging challenges, refine existing methodologies, and improve the effectiveness of precision medicine in cancer care. The tools discussed herein offer a snapshot of the current state of the field but are designed to evolve. Bioinformaticians, staying abreast of the constantly changing technologies and tools, play a central role in the realm of precision oncology.

The application of deep learning methods in the field has only just begun, with AlphaMisense serving as an illustrative example of how this technological gap is starting to revolutionize various aspects of data analysis, including bioinformatics. The next phase of developments will likely involve the application of advanced AI algorithms to aligners and variant callers. While reference genomes are evolving, aligners have remained unchanged for several years and are due for an update. Additionally, DeepVariant [88], a deep learning-based variant caller currently not applied to somatic variant calling, is expected to be adapted to this specific case in the coming years.

Moreover, while tumor mutational burden (TMB), representing the total count of DNA mutations detected in cancer cells and an important biomarker for immunotherapy [89,90,91], traditionally relied on whole genome sequencing or whole exome sequencing, it can now be estimated through targeted sequencing of a focused gene panel [92]. However, a recent study by Fang et al. [93] revealed that panels focusing on cancer genes tend to overestimate TMB in comparison to whole exome sequencing. This overestimation is mainly due to the positive selection for mutations in cancer genes. While the complete resolution of this issue remains elusive through the removal of mutational hotspots alone, a meticulous calibration process can enable a truthful TMB calculation within a clinical context. Its seamless integration into somatic pipelines is anticipated in the near future.

Of particular significance is also the development of a user-friendly interface essential to ensure accessibility and effective analysis by physicians of the outcomes yielded by the delineated pipeline, including the genotyping results, the sequencing quality metrics, and the run quality metrics. To our knowledge, no reports of a tool offering this type of interface have been published, and additional work seems necessary to create one. The genotyping results from the various analyses are aggregated to provide a comprehensive overview of the patients’ genotype. This aggregation facilitates precision medicine approaches by offering a holistic understanding of individual genetic profiles. Additionally, the sequencing run metrics furnish insights into diverse aspects of the sequencing process, encompassing the quantity of generated reads, read length, read quality, and the coverage level. They thus offer the opportunity to evaluate the performance of the sequencing apparatus and the caliber of the generated sequencing data. Through careful examination of these metrics, both bioinformaticians and physicians can detect potential issues that might affect data quality. Subsequently, this information can be exploited to optimize sequencing conditions, potentially conduct a re-run if warranted, or adapt downstream analysis methodologies to account for identified issues. While such reports thus play essential roles in important patient management decisions, they are often overlooked. Such an interface would need to meet the specific needs of laboratory-based physicians analyzing several thousand samples annually. Its development would thus require the close collaboration between bioinformaticians and physicians.

## 7. Conclusions

Access to dependable bioinformatics pipelines is imperative in precision oncology. They facilitate the accurate identification and interpretation of genomic alterations on which treatment decisions are based (Table 2). However, bioinformatics pipelines often entail computationally intensive steps, often requiring high-performance computing clusters or robust cloud computing resources. Such computational demands must be meticulously considered by bioinformaticians and medical staff when planning to implement such an approach, as poorly designed architecture can result in delays in obtaining results or, in some cases, a failure to obtain any results. It is noteworthy that private solutions such as Sentieon [94] or NVIDIA Parabricks [95] propose to accelerate large-scale data analyses, resulting in overall pipeline execution time savings ranging from three- to eightfold [96].

A well-designed and well-documented bioinformatics pipeline provides reliable and accurate guidance for oncologists, ultimately leading to better outcomes for patients. Variant calling, interpretation, and annotation represent critical steps in precision oncology, and rely on bioinformatics expertise and technology. They are altogether aimed at providing personalized cancer treatment dependent on the tumor-specimen-specific genetic alteration revealed.

Variant calling is a complex and challenging task due to the high levels of background noise and variation present in NGS data, as well as the need to distinguish true cancer-related alterations from germline or benign variants. To address these challenges, advanced bioinformatics tools and algorithms have been developed that exploit various strategies, such as statistical modeling or machine and deep learning, to improve the sensitivity, specificity, and reproducibility of variant calling.

Once the genomic variants have been called, the next step is to annotate and interpret them in the context of known biological and clinical knowledge. This includes identifying the functional impact of the variants on proteins and related biological pathways, as well as assessing their potential relevance to cancer development and treatment. In this context, bioinformatics resources such as public databases, biological pathway analysis tools, and drug–gene interaction databases are indispensable to prioritize and contextualize the genomic findings.

By integrating multiple sources of genomic and clinical data, bioinformatics can help identify the most relevant molecular targets and therapeutic options for cancer patients, ultimately improving their outcomes and quality of life. A crucial step in precision oncology is the clinical reporting of molecular findings, which involves the translation of complex genomic data into meaningful clinical implications that can guide patient care. The clinical report should provide clear and concise information on the identified molecular alterations, their relevance to the patient’s disease, potential therapeutic options, and any relevant clinical trials. It should also highlight any issues in the data, as well as provide recommendations for further testing or monitoring. The entire process must ensure that the clinical report accurately reflects the molecular landscape of the patient’s disease and provides actionable information to guide personalized treatment decisions.

## Figures and Tables

**Table 2:** Latest NGS DNA analyses recommended by international guidelines. ESMO: European Society for Medical Oncology; NCCN: National Comprehensive Cancer Network; EANO: European Association of Neuro-Oncology; ESGO: European Society of Gynaecological Oncology; ESTRO: European SocieTy for Radiotherapy and Oncology; ESP: European Society of Pathology.

Tumor Type	Alterations	Guidelines	References
Metastatic non-small-cell lung cancer	*EGFR* exons 18–21 mutations	ESMO 2023	[97]
	*BRAF* V600 mutation		
	*NTRK* rearrangement		
	*KRAS* G12C mutation		
	*HER2* exon 20 mutations		
	*MET* exon 14 skipping mutations		
	*MET* amplifications		
Cutaneous melanoma	*BRAF* V600 mutation	NCCN 2023	NCCN Guidelines Version 2.2023 Melanoma: cutaneous
	NRAS G12, G13, Q61 mutations		
	KIT Exons 8, 9, 11, 13 and 17 mutations		
High-grade glioma	*IDH1* R132 mutation	EANO 2021	[98]
	*IDH2* R172 mutation		
	*TERT* promotor mutation		
	*TP53* mutations		
	Histone H3 K27M mutations		
	*CDKN2A/B* deletions		
	*EGFR* amplification		
High-grade serous ovarian cancer	*BRCA1* and *BRCA2* mutations	ESMO 2019	[99]
	Homologous recombination deficiency		
Endometrial carcinoma	*BRAF* V600 mutation	ESGO/ESTRO/ESP 2021	[100]
	*POLE* mutations		
	*TP53* mutations		
	Microsatellite instability		
Metastatic colorectal carcinoma	KRAS Exons 2–4 mutations	NCCN 2023	NCCN Guidelines Version 3.2023 Colon Cancer
	NRAS Exons 2–4 mutations		
	*BRAF* V600 mutation		
	Microsatellite instability		
	*HER2* amplification		
Thyroid carcinoma	*BRAF* V600 mutation	NCCN 2023	NCCN Guidelines Version 4.2023 Thyroid Carcinoma
	*RET* mutations		
GIST	*KIT* Exons 8–11 mutations	ESMO 2022	[101]
	*PDGFRA* D842V mutation		
Pancreatic adenocarcinoma	Microsatellite instability	NCCN 2023	NCCN Guidelines Version 2.2023 Pancreatic adenocarcinoma
	*KRAS* G12C mutation		
	*BRCA1* and *BRCA2* mutations		
	*BRAF* V600 mutation *		

* Not included in European guidelines to date.

## Data Availability

Not applicable.
